# Conceptual reflection on affirmative nursing as a professional field for the care of older gay men

**DOI:** 10.1590/1980-220X-REEUSP-2025-0517en

**Published:** 2026-04-10

**Authors:** Vinicius de Oliveira Muniz, Pricila Oliveira de Araújo, Evanilda Souza de Santana Carvalho, Rosalina Aparecida Partezani Rodrigues, Anderson Reis de Sousa

**Affiliations:** 1 Universidade Federal da Bahia, Escola de Enfermagem, Salvador, BA, Brazil.; 2 Universidade Estadual de Feira de Santana, Feira de Santana, BA, Brazil.; 3 Universidade de São Paulo, Escola de Enfermagem de Ribeirão Preto, Ribeirão Preto, SP, Brazil.

**Keywords:** Men’s Health, Health of the Elderly, Nursing, Sexual and Gender Minorities, Public Policy

## Abstract

**Objective:**

To reflect on Affirmative Nursing care as support for the expression of sexual orientation in older gay men.

**Method:**

A theoretical study, developed between 2023 and 2025, including articles, government materials, observatories, a manual, a book/book chapter, and a resolution. The analysis was conducted through interpretive reading, based on the framework of Affirmative Action, guided by a checklist. Development: Heteronormativity reflects a problematic scenario that such men experience worldwide, surrounded by homophobia, symbolic violence, marginalization by health services due to embarrassing experiences, self-segregation, double stigmatization, social exclusion, and invisibility, contributing to an impaired Coming Out. Affirmative Nursing is based on principles of affective, inclusive, and culturally sensitive care. Together with social gerontology and public policies, they propose practices that recognize identities, promote safe environments, and strengthen therapeutic bonds.

**Conclusion:**

Creating a caregiving trend integrated into affirmative action policies is a way to reduce prejudice and increase adherence to health services, a practice that needs to be configured to safeguard the dignity of older gay men, protecting them from any inhumane and/or embarrassing treatment.

## INTRODUCTION

Older gay men have been socially pressured to think of sexuality as a mental illness, a health deviation or immorality, which has led most of them to live their homosexuality believing that heterosexuality is something superior and the only way to express sexual orientation^(1)^. Added to this are experiences of ongoing discrimination, the HIV/AIDS crisis, and challenges in coping with the complexities of aging^(2)^. This contributes to the need to support healthcare from an Affirmative Nursing perspective, based on initiatives that suspend methods of social exclusion, validate diversity, and celebrate identities by recognizing them as strengths^(3,4)^.

The “Affirmative Nursing” approach argues through a dialogue close to the constructs of affirmative care^(4)^, of affirmative policy^(5)^, and affirmative action^(3)^, due to configurations focused on strategies to combat inequality based on sexual orientation, using, in clinical practice, instruments of historical reparation, anti-discrimination and/or compensation for disadvantages arising from structures, behaviors and attitudes^(3,4)^. As a practice, it demands systematic and scientific development from a political and ethical perspective, as it challenges cisheteronormative standards prevalent in health services in general. Therefore, it deals with overcoming the lack of theoretical direction in practice, the scarcity, and the emptying of meaning and professional purpose when meeting the individuals’ unique demands and needs.

Double discrimination based on age and sexual orientation can exemplify a scenario in which intersectionality occurs and multiple marginalized identities intersect, substantiating social harm and vulnerabilities while inequalities and oppressions are amplified^(6)^. Additional factors such as skin color and socioeconomic status tend to exacerbate these inequalities, potentially revealing that black older gay people present critical indicators of access to healthcare within this group, which contributes to feelings of guilt, sadness, humiliation, and social burden^(7,8,9)^.

In addition, it is observed that society fosters fear of old age by associating it with illness, exalts youth and marginalizes the older people, as well as denying them the right to exercise their sexuality, suggesting that intersectional classes are present in the daily lives of these people. When it comes to older gay men seeking health services, they often conceal their sexual orientation, which is characterized as an Impaired *Coming Out* ^(10)^. In other words, remaining “in the closet” as a strategy to cope with prejudice, from the perspective of protecting identity – preserving the self – but which consequently contributes to low self-esteem, social exclusion, repression, and isolation. Such insecurity can lead to unhealthy transitions during life cycles and aging.

Studies regarding care for older men have underexplored gay men’s health, both in the specific field of Nursing and in the interdisciplinary fields of geriatrics and gerontology, demonstrating a unidirectional and reductionist approach to only some LGBTQIAPN+ representations^(11)^. This gap impacts the healthy experience of sexuality and the safe expression of homosexuality, a fact also reflected in the guidelines of the National Health Policy for the Older People^(12)^ and in the Care Guide for the Older People^(13)^, both published in Brazil, as they briefly address the issue of same-sex aging, without pointing to ways to systematically plan/manage care needs.

This invisibility is reflected in studies on violence. Although the United States (USA) has recorded that 17.2% of 14,243 victims of hate crimes were motivated by sexual orientation, including homicides, assaults, and other types of violence, there is no specific data on older gay men^(14)^. In Brazil, there were 165 homicides of gay men in 2024, without specifying an age range. In 2019, 3.9% of LGBT+ deaths were of older gay men between 68–70 years of age, in the northern and northeastern regions of Brazil. Ceará, São Paulo, and Pernambuco are the states with the highest number of victims^(15)^.

Despite constituting a significant proportion of violent events, there is no reliable global aggregate number available that includes sexual orientation in death data systems in many countries, nor is there official data collection due to the criminalization of same-sex relationships, for example, in Asia and Africa. This contributes to fragmented reporting and the lack of long-term awareness strategies^(16)^, since the political system itself is discriminatory in these regions, drawing attention to healthcare provided in primary care, hospitals, clinics, home care, specialized centers, rehabilitation facilities, and other settings.

These are underreported figures that lead to the reflection that the persistence of violence against this group reinforces the urgency for effective, protective, and compassionate public policies to combat hate crimes related to homophobia. As can be observed, homicide ranks first among causes of death and suicide is second, reflecting a distorted and prejudiced view of old age and homosexuality, contributing to the onset of psychological suffering^(9,17)^. This requires a repositioning of healthcare professionals, from the perspective of affirming sexually diverse identities.

In 2022, in Brazil, 13% of the world’s population were older people, equivalent to 1.1 billion individuals^(18)^, with 14 million older men having a life expectancy of 73 years, seven years less than women^(5,19)^. This is evidence that care is unequal and that the fragmented nature of gender equality needs to be overcome. Globally, this difference is somewhat reduced, but men still live about 4.8 years less. The National Health Survey^(20)^, in 2019, recorded 159.2 million inhabitants over 18 years of age; of these, 46.8% are men and 1.9% self-identified as gays. It is observed that the older the age group, the lower the rate of self-reporting of sexual orientation, suggesting that the public prefers to conceal this information due to insecurity in acknowledging their homosexuality.

Concealing information about sexual orientation may be related to a self-preservation strategy in the face of perceived stigma, which anticipates the discrimination to which they are subject in spaces of interaction with health professionals. However, while protecting the deteriorated identity, concealment prevents the advancement of collective actions by the group because it renders it invisible.

Hypotheses point to the idea that the older gay men’s health is not a priority in collective and individual actions^(21,22,23)^ due to sociopolitical failures and disparities in the fulfillment of the principles of equality in relation to the heterosexual public^(23)^.

The intention here is to provide a focused reflection on the social, political, and health landscape, offering a range of information about the male gay population faced with the challenges and obstacles associated with aging and access to healthcare services, which often force them to conceal their identity^(24)^ and, most importantly, providing theoretical and guiding material for thinking about affirmative practices during interdisciplinary healthcare services, with the aim of advancing the implementation of policies that seek to provide humane care for the older people and their specific needs. Given this problem, the objective was to reflect on Affirmative Nursing care as support for the expression of sexual orientation in older gay men.

## METHOD

This theoretical-reflective essay, focused on the context of social protection and well-being of the target audience, was developed from 2023 to 2025. It is a product of the first author’s doctoral dissertation entitled “Older and cisgender men and homoaffectivity: from technological prospecting to a proposition for affirmative nursing and health practice,” and is guided by the following question: what are the theoretical reflections that lead to an understanding of Affirmative Nursing care as support for the expression of sexual orientation in older gay men aimed at transitioning to healthy aging?

This issue allowed for a more in-depth narrative approach and the identification of the attributes of the research object, through the selection of 47 published materials, including: 17 national and 13 international scientific articles, via *SciELO* and *PubMed/MedLine,* seven national government materials, a resolution from the Federal Nursing Council, statistical data from an observatory, three national and two international statistical data materials, one international manual, one national book and one national book chapter found in *Google Scholar* and in official sites. The following descriptors were used: Men’s Health, Older People Health, Nursing, Sexual and Gender Minorities, and Public Policy. The research team has backgrounds in Nursing, Public Health, and Gerontology, as well as experience in research on male homosexuality.

Materials were included that addressed elements of the object of interest, such as the phenomenon of gay men aging and nuances related to social aspects, public policies, forms of care, relationships between homosexuality and repercussions on biopsychosocial health, materials without time limit, in Brazilian Portuguese, Spanish and English (authors’ languages). Corrupted files have been deleted.

The analysis was conducted through an interpretative reading based on the political framework of Affirmative Action^(3)^, guided by a checklist created by the authors containing key elements such as: does the text describe social and political micro-phenomena related to older gay men, even including other sexual minority audiences? Does the content describe public policies focused on gay men’s health in a gerontological context? Does the material address difficulties in access, health problems, and care from the perspective of affirmative action? Ethical considerations were respected with a view to ensuring the reliability, veracity, and quality of the data produced/analyzed.

## DEVELOPMENT

### Health and Illness in Old Age Among Gay Men and Affirmative Nursing as a Field For Guaranteeing The Right to Care

Affirmative Nursing, grounded in principles of culturally sensitive care, along with social gerontology, proposes practices that promote safe environments and strengthen therapeutic bonds, aligning with public health policies for gay men, aiming at greater healthcare adherence^(7,13,25,26,27)^. It is worth highlighting that affirmative and socially supportive approaches can be crucial for subjective well-being and social integration by offering support mechanisms that mitigate the deleterious effects of long-term discrimination by reducing psychosocial suffering^(2)^.

Throughout the 21st century, the expansion of institutional and symbolic spaces for debate about the gay population in the field of health has fostered greater social visibility. This movement, driven by intersectoral policies in the areas of culture, education, labor, health, social security, and justice, strengthened the agenda for combating homophobia and consolidated comprehensive health care as a structuring axis of affirmative actions^(7)^. Therefore, the trend of Affirmative Nursing is believed to be a driving force in facilitating the guarantee of rights and social justice, through the recognition and valuing of the socially referenced person, just as in advocacy. However, even with regulatory advances, the delivery of care remains unequal and requires the instrumentalization of professional practices to respond to emerging demands^(10)^.

In the Brazilian context, institutional milestones such as the program *Brazil without Homophobia* (2004), the 1st National LGBT+ Conference (2008), and the incorporation of LGBT+ policy by the National Health Council (2011) constituted important advances in the articulation between sexual rights and public health policies, including dialogue with men’s health, older people’s health, and mental health policies^(7,13)^. However, the existence of these devices does not, in itself, guarantee the transformation of daily care practices, reinforcing the need for care models that incorporate affirmative action as a structuring axis of expanded clinical practice.

Discriminatory experiences, both the recent and lifelong ones, predict greater psychological distress and poorer self-perception of health among older gay men^(17,28,29)^. This corroborates the idea that inclusive policies, when not accompanied by changes in care practices, produce limited impacts on reducing health inequities. In this context, the following question is raised: are the conduct, position, and professional care of nursing professionals culturally competent and congruent with the individuals’ sexual and gender diversity?

The prevailing focus in neoliberal approaches to public health prioritizing personal responsibility and considering gay men’s sexual practices as a risk, coupled with a lack of understanding of how these factors promote and protect health more comprehensively, reflects the insufficient attention given to the social, economic, and personal contexts of life and to the intersections between context and behavior, since homophobia is a proven contributing factor to health inequalities and is reflected in ineffective health management^(30)^, because if the system fosters prejudice in a veiled way, the advancement of affirmative action is stalled, since the system consists of people.

Affirmative Nursing, consequently, aims to respect the expression of people/patients, whether in the context of sexual orientation or gender identity, using resources that employ inclusive language, vocal adaptation, understanding of terminology, respectful care, the creation of safe and creative environments, communication centered on the person and their subjectivity, and the avoidance of assumptions and compulsory norms. Furthermore, it involves understanding the culture of these men, the systemic changes, and the existing transition processes.

Understanding these continuously evolving processes is the first step towards the development of customized resources that respect socio-historical contexts while simultaneously seeking to overcome current challenges. Essentially, the detrimental effects of abandonment have to be recognized and addressed simultaneously for better healthcare^(31)^, aimed at building trusting relationships and reducing health disparities.

The vulnerability experienced by this community is characterized by unique minority stressors attributed to intersectional factors such as age, ableism, ethnicity, employment status^(32)^, and the main area of interest, homosexuality, because despite constituting a structuring dimension of human sexuality related to how individuals experience affective-romantic and sexual bonds with people of the same gender^(4,7)^ it is still far from being respectfully integrated with fundamental human rights and equal freedom of expression, with security before the law and protection against oppressive practices^(1,7,33)^. This aligns with cases of internalized homophobia, where individuals begin to internalize the repulsion associated with homosexuality and construct a negative self-concept of their own sexual orientation, requiring coping strategies and self-acceptance.

It is worth noting that society views older people in isolation from issues of sexuality, and when the subject comes up, another exclusionary factor emerges: destructive and perverse criticism of inappropriate behavior based on the social logic that old age should be exempt from enjoying life pleasures and intimate satisfactions. In society, asexual representations of old age are disseminated, which intersect with exacerbated and deviant sexual behaviors. These ideas foster expectations that older people in general should give up desire, pleasure, and intimacy with their peers. An affirmative approach to care considers the need to invest in the circulation of ideas and images that challenge this scenario that denies sexuality and imposes control over aging bodies.

Therefore, it becomes crucial to connect aging to the intersectionality of age, gender, and sexual orientation, as these are embedded in discriminatory processes, recognizing the experiences of health trajectories marked by cumulative inequalities, making affirmative action in health a strategy for creating personalized interventions sensitive to lived traumas^(2)^.

With the existence of fragments in the pursuit of equity that lead this population to face structural disparities in access to health services and social support, conditioned by stressors accumulated throughout life, such as a lower probability of being married and having a family^(27)^, the disparity between the formal existence of rights and the actual experience of caring and compassionate care is widened^(13)^.

Meanwhile, there is an increased risk of development of diagnoses such as depression and anxiety compared to heterosexual men, as well as serious psychological disorders as a consequence of inadequate emotional support and unequal community treatment, exacerbated by interpersonal discrimination^(2)^. This outlines opportunities to strengthen protective factors such as economic security and social support, mitigating the risk of illness^(27)^, since affirmative approaches reduce psycho-emotional symptoms by promoting recognition and relational security^(3)^.

Social and institutional challenges highlight experiences of loneliness and social invisibility that are detrimental to the aging process^(2,8)^, leading to embarrassing experiences during healthcare appointments in global contexts. This situation requires healthcare professionals to acknowledge the reality and focus on mitigating disproportionate care by adopting strategies that promote the affirmation of sexual orientation, as well as a psychosocial, cultural, and spiritual approach for holistic and compassionate care^(32)^, with the possibility of addressing fragmented engagements in preventive and long-term care.

Violence, including its symbolic forms considered “jokes”, is a relevant social marker in this context, sustained by heteronormative structures that value certain arrangements and devalue dissident sexual orientations by considering heterosexuality superior, the only normal, natural, correct, and valid one because it follows a dominant hegemonic model^(22,34)^. Homosexuals are treated with derogatory terms such as *“aunts who do* [sexual intercourse] *the teenager”, “Susana Vieira fags”, “old queens” and “decrepit queens”,* leading to the reflection that they are not abject or immoral beings, but rather ethical and moral citizens who need to reinvent themselves each day, since gay old age is considered to be rejected by the heterosexist culture^(35)^. Hence, Affirmative Nursing turns to correcting structural differences and achieving social recognition^(3)^.

The existence of labeling language, excruciating, discourteous, violent and negligent attitudes/practices (from society and caregivers) has to be considered and, based on this, a vigilant stance towards the public being assisted has to be adopted. We propose that a first step be openness to dialogue, with sensitive listening to what the group expects from care, their complaints, criticisms, and suggestions; training guided by theories on gender, sexuality, and human rights; less adherence to models focused on clinical complaints; and support for holistic care models that place the person and their history as the axis/focus of the encounter. Given this, in the Brazilian context, affirmative action has become established as an instrument of social justice throughout the redemocratization process, guiding public policies aimed at inclusion and equity^(3,4)^.

The combination of affirmative structural and mental health care can acknowledge cumulative negative experiences and lead to improvements in health, within the logic of its expanded concept, always seeking to encourage reflection by professionals on how to reorganize their services, whether in primary care or any other sector, always remembering that the population has the right to be treated free from any type of prejudice and that any exclusion based on age, sexual orientation, and other intersecting categories is prohibited^(7,36)^.

This idea of reorienting healthcare practices is due to the frequent behavior of reducing the gay population to specific organic markers that mask other demands related to aging, subjectivities, and social relations, and that, in some realities, it is assumed that the family structure is only heterosexual and cisgender, thus rigidifying care that does not, initially, consider the diversity of family units^(7,21,22,33,37)^.

The lack of connection between the sociocultural dimension of demerit and the care provided in healthcare facilities undermines trust and intensifies the feeling of not knowing what to do or where to go when help/support is needed, reflecting a pattern that resonates in different international contexts and has to be overcome^(10,24)^. Added to this is the association between aging, an increase in potential biopsychological health problems, and leading a precarious life with disabilities—factors that are critical for maintaining quality of life.

It is recommended to reflect on the assistance provided in light of the failure of the biomedical model, when looking at the gay population and viewing only clinical and pathological aspects related to the sexual apparatus in a misguided and negligent way, and to acknowledge that the social environment and human interactions have implications for health outcomes in old age (community, friends, family, work, among others), by focusing on the apparent neutrality of the biological view, which incorporates sensitive and dialogical approaches^(3,4)^, makes access easier^(33)^, and recognizes that formal universality does not eliminate institutional barriers, since change generally occurs in the process of self-awareness anchored in the principle of material equality, the valuing of differences, the promotion of same-sex citizenship, the strengthening of social protection networks, and real balance^(3,4,5,7,13,38)^, breaking, in a way, with the privilege-oppression dichotomy.

National guidelines that guide comprehensive care, such as the work “Gay and Bisexual Men: Rights, Health, and Social Participation”, the outcomes of the 2nd National LGBT+ Conference^(7)^ and the recommendations in the “Guide to Care for the Older Person”^(13)^, especially with regard to Comprehensive Geriatric Assessment (CGA), are possible implementation resources. CGA, in particular, because it is an approach that goes beyond the physical body, allows for a diagnosis of more existential layers, including for those in situations of socioeconomic vulnerability. Older people with geographical limitations can also benefit from Telehealth^(39)^, reducing barriers related to misinformation and the unequal provision of services^(4,40)^ by expanding the possibilities of access to affirmative care.

These guidelines, when combined with nursing consultations and shared therapeutic planning, promote functional rehabilitation practices, with effective commitments agreed upon between professionals, users, and services^(1,41,42)^ as it is a model that recognizes individual differences and promotes autonomy and active participation in care. From an ethical and legal standpoint, Affirmative Nursing finds support in the Code of Ethics for Nursing Professionals^(43)^ and in the Bill of Rights of Health Service Users^(44)^, which ensure respect for dignity, freedom of expression of sexual orientation, provision of care free from discrimination, and the maintenance of hope.

Thus, the inclusion of the depathologization of homosexuality in professional activities, the analysis of corporalities and functional diversity in aging, as well as the creation of collective spaces – in person or virtual – for sharing experiences, are coping strategies for promoting psychosocial well-being^(5,29)^. These elements constitute a care approach built collectively and interdisciplinarily, aligned with contemporary propositions of gerontological nursing, and which can contribute to the decongestion of traditional public health services in a sustainable and intelligent way.

Regarding the assumptions of public health, nursing, along with other disciplines such as psychology, social work, and medicine, needs to focus on recording and giving visibility to the individuals’ narratives, valuing their memories and experiences, as opposed to imposing a single way of being or relating. It should include everyone in broader debates about the importance of staying updated and respecting what is plural^(45)^, exploring experiences and ways of being a man while being a gay man and rethink the stance of resistance and uniqueness within a social context that often imposes harmful and exclusionary standards^(46)^, recognizing that sexual diversity transcends fixed classifications and its manifestation relates to subjective individual issues.

The study of sexual diversity needs to be fully integrated into gerontology, as emphasizing the important roles of self- affirmation and autonomy contributes to the recognition of human rights as essential in promoting sexual life quality^(29)^. Consequently, the caregiving concept addressed in this essay is configured as an emerging and strategic field that articulates the production of care with different fields/disciplines that interconnect in practice, because by recognizing the uniqueness of life trajectories, masculinities, and experiences of aging, professionals will contribute to reducing inequalities, strengthening citizenship, and building therapeutic itineraries that are sensitive not only to clinical dimensions.

## POTENTIALITIES AND IMPLICATIONS FOR NURSING PRACTICE

This is a phenomenon that is still little explored by nursing, and the production of data on a historically excluded group can stimulate the strategic implementation of models in intercultural spaces^(3)^, capable of strengthening the recognition of Affirmative Nursing as an equitable clinical-social and professional field that implements interventions focused on overcoming trauma by addressing underlying issues that help cultivate supportive, social relationships, of resilience and positive identity factors, as they are significant predictors of well-being and successful aging^(47)^.

It is known that understanding the configuration of resources and risks by age group and sexual orientation is important for the development of health initiatives adapted to the recognition of the systemic role of discrimination, while also supporting individual efforts in the active management of health, taking into account the heterogeneity in the understanding of sexuality and its dimensions in old age.

These elements can be developed during the provision of affirmative care, and what emerges from these reflections is the idea that the interdisciplinary field working in the area of health needs to have a practical and sensitive perspective to overcome interconnected systems of oppression through meaningful social connections, such as fostering authentic and deep relationships with the purpose of cultivating longitudinal and anti-stigmatizing follow-up^(2)^ focused on promoting support for the expression of sexual orientation.

To strategically support the idea of improving care, it is relevant to provide culturally relevant training for healthcare professionals through unified training programs in terms of duration, content, and methodology, especially when key terms/terminology, sexuality, sexual history (dysfunctions), and the specific health of older gay men and health disparities are addressed. This has resulted in improved short-term knowledge among this audience regarding the needs of older men who are gay, always including them in continuing education programs.

The advancement of knowledge regarding the diversity of sexual orientation and age, and the structuring of a new care model with perspectives on broader approaches in the hiring of male gay nurses*,* with the intention of forming teams that can develop the individual’s autonomy and reflect on a more effective approach and exchange of ideas, are points to consider within institutions, as is the encouragement to offer specific disciplines in undergraduate and graduate programs from a more diverse, fluid, and relevant curricular perspective, given that education is still focused on heterosexuality.

## CONCLUSIONS

Despite the progress made in affirmative action policies and existing guiding documents in the Brazilian context, obstacles to the effectiveness of Affirmative Nursing care as a support to the expression of sexual orientation of older gay men stem from a combination of negligence, deficiencies, and absences in the practical field of care. The absence of inclusive approaches in curricula, the silence surrounding sexuality, and the taboos related to the bodies of older people justify the idea of creating a care-oriented approach integrated into affirmative action policies as a way to reduce social and institutional prejudice and increase these men’s adherence to health services.

Practices that are consistent with the needs of these men must be designed to safeguard their dignity throughout all stages of development, protecting them from any inhumane, violent, terrifying, humiliating, or embarrassing treatment as they age.

It is expected that, in the future, a more structured and institutionalized technical and scientific systematization will be possible so that the clinical judgment of problems that elicit human responses can be better executed and enable healthy transitions of these social actors with mastery. Ultimately, taking care of the health of older gay men is possible based on the comprehension of their needs, not just fulfilling duties, but also on a collective understanding of the importance of providing protection.

Nurses need to develop studies focused on understanding older gay men’s health, as this fact has limited the discussion of this production in a more focused way, since most studies are generalized and involve the entire population of sexual, gender, gender identity, and gender expression minorities. Therefore, it is necessary to define the target audience, as the specific characteristics tend to differ, as well as to conduct research that measures mental health variables and social challenges related specifically to male homosexuality, since these are interconnected themes.

## DATA AVAILABILITY

All the data supporting the results of this study were published in the article itself.
