# Supplementary material for: Conceptual reflection on affirmative nursing as a professional field for the care of older gay men
Source: Rev Esc Enferm USP. 2026 Apr 10;60:e20250517. doi: 10.1590/1980-220X-REEUSP-2025-0517en (PMC13090036; doi:10.1590/1980-220X-REEUSP-2025-0517en)
Supplement: Translation

# Translation: Reflexão conceitual da Enfermagem Afirmativa como um campo profissional para o cuidado de homens idosos *gays*

**DOI:** 10.1590/1980-220X-REEUSP-2025-0517pt

**Authors:** Vinicius de Oliveira Muniz, Pricila Oliveira de Araújo, Evanilda Souza de Santana Carvalho, Rosalina Aparecida Partezani Rodrigues, Anderson Reis de Sousa

**Affiliations:** 1 Universidade Federal da Bahia, Escola de Enfermagem, Salvador, BA, Brasil.; 2 Universidade Estadual de Feira de Santana, Feira de Santana, BA, Brasil.; 3 Universidade de São Paulo, Escola de Enfermagem de Ribeirão Preto, Ribeirão Preto, SP, Brasil.

**Keywords:** Saúde do Homem, Saúde do Idoso, Enfermagem, Minorias Sexuais e de Gênero, Política Pública

## Abstract

**Objetivo:**

Refletir sobre os cuidados de Enfermagem Afirmativa como apoio à expressão da orientação sexual de homens idosos *gays*.

**Método:**

Estudo teórico, desenvolvido entre 2023/2025, incluindo artigos, materiais governamentais, observatórios, manual, livro/capítulo de livro e resolução. A análise foi realizada por meio da leitura interpretativa, baseada no referencial da Ação Afirmativa, guiada por *checklist*.

**Desenvolvimento:**

A heteronormatividade reflete um cenário problemático que tais homens vivenciam mundialmente, circundado de homofobia, violência simbólica, marginalização pelos serviços de saúde devido a experiências constrangedoras, autossegregação, dupla estigmatização, exclusão social e invisibilidade, contribuintes para um *Coming Out* prejudicado. A Enfermagem Afirmativa fundamenta-se em princípios de cuidado afetivo, inclusivo e culturalmente sensível. Juntamente da gerontologia social e de políticas públicas, propõem práticas que reconhecem identidades, promovem ambientes seguros e fortalecem vínculos terapêuticos.

**Considerações finais:**

Criar uma tendência cuidativa integrada à política de ação afirmativa é uma forma de reduzir o preconceito e aumentar a adesão aos serviços de saúde, prática que precisa ser configurada para zelar a dignidade do homem idoso *gay*, preservando-o de qualquer tratamento desumano e/ou constrangedor.

## INTRODUÇÃO

Homens *gays* idosos foram pressionados socialmente a pensar a sexualidade como doença mental, desvio de saúde ou imoralidade, o que tem levado a maioria deles a viver a sua homossexualidade acreditando que a heterossexualidade seria algo superior e a única forma de expressar a orientação sexual^(1)^. Somam-se a isso as experiências de discriminação contínua, crise do HIV/aids e desafios para lidar com as complexidades do envelhecimento^(2)^, o que contribui para a necessidade de fortalecer o cuidado em saúde, na perspectiva da Enfermagem Afirmativa, baseada em iniciativas de suspensão de métodos de exclusão social que validem a diversidade e celebrem as identidades ao reconhecê-las como pontos fortes^(3,4)^.

A abordagem da “Enfermagem Afirmativa” argumenta por meio de um diálogo próximo aos construtos do cuidado afirmativo^(4)^, da política afirmativa^(5)^ e da ação afirmativa^(3)^, devido a configurações voltadas a estratégias de combate à desigualdade por orientação sexual, utilizando na prática clínica instrumentos de reparação histórica, antidiscriminatórios e/ou de compensação de desvantagens decorrentes das estruturas, comportamentos e atitudes^(3,4)^. Enquanto prática, demanda o desenvolvimento sistemático e científico sob perspectiva política e ética, pois desafia normas cisheteronormativas prevalentes nos serviços de saúde de modo geral. Destarte, ocupa-se com a superação da falta de direcionamento teórico do fazer, da escassez e do esvaziamento de sentido e propósito profissional no atendimento a demandas e necessidades singulares dos sujeitos.

A dupla discriminação por idade e orientação sexual pode exemplificar um cenário no qual a interseccionalidade ocorre e em que múltiplas identidades marginalizadas se cruzam, o que substancia agravos e vulnerabilidades sociais, na medida em que desigualdades e opressões se amplificam^(6)^. Fatores adicionais como cor de pele e condição socioeconômica tendem a potencializar essas desigualdades, com capacidade para talvez, revelar que pessoas idosas *gays* negras apresentam indicadores críticos de acesso à saúde dentro desse coletivo, o que contribui para o sentimento de culpa, tristeza, humilhação e fardo social^(7,8,9)^.

Aliado a isso, observa-se que a sociedade fomenta o medo da velhice ao associá-la à doença, exalta a juventude e marginaliza a pessoa idosa, além de negar-lhe o direito de exercer sua sexualidade, tendenciando que classes interseccionais estão presentes no cotidiano destas pessoas. Quando se trata de homens idosos *gays* que procuram os serviços de saúde, esses ocultam a orientação sexual, o que se caracteriza como um *Coming Out* Prejudicado^(10)^, ou seja, permanecer “dentro do armário” como estratégia para o enfrentamento do preconceito, na perspectiva da proteção da identidade – preservação do eu, mas que consequentemente contribui para baixa autoestima, despertencimento social, repressão e isolamento. Tal insegurança pode implicar em transições insalubres durante os ciclos vitais e n o envelhecimento.

Estudos acerca dos cuidados voltados a homens idosos têm explorado pouco a saúde dos homens *gays*, tanto no campo específico da Enfermagem quanto no interdisciplinar da geriatria e gerontologia, demonstrando unidirecionalidade e reducionismo de apenas algumas das representações LGBTQIAPN+^(11)^. Essa lacuna implica na vivência saudável da sexualidade e na expressão com segurança da homossexualidade, que também é percebida nas diretrizes da Política Nacional de Saúde da Pessoa Idosa^(12)^ e no Guia de Cuidados para a Pessoa Idosa^(13)^, ambos publicados no Brasil, pois estes abordam resumidamente a questão do envelhecimento homoafetivo, sem apontar caminhos para um planejamento/manejo sistematizado das demandas de cuidados.

Essa invisibilidade é refletida nos estudos sobre violência. Embora os Estados Unidos (EUA) tenham registrado que 17,2% de 14.243 vítimas de crimes de ódio foram motivados por orientação sexual, dentre os quais homicídios, agressões e outros tipos de violência, não há dados específicos de homens idosos *gays* ^(14)^. No Brasil, ocorreram 165 homicídios de homens *gays* em 2024, sem especificidade de faixa-etária. Em 2019, 3,9% das mortes LGBT+ foram de homens idosos *gays*, entre 68–70 anos de idade, nas regiões brasileiras norte-nordeste. Ceará, São Paulo e Pernambuco são os estados com o maior número de vítimas^(15)^.

Apesar de constituir uma proporção significativa dos eventos violentos, não há um número agregado mundial confiável disponível que inclua orientação sexual nos sistemas de dados de óbito em muitos países, nem coleta oficial de dados devido a criminalização de relações homoafetivas como, por exemplo, nos continentes asiático e africano, o que contribui para a fragmentação das notificações e para o não fortalecimento de estratégias de conscientização em longo prazo^(16)^, uma vez que o próprio sistema político é discriminatório nessas regiões e faz despertar atenção direcionada ao cuidado em saúde produzido na Atenção Primária, hospitais, ambulatórios, Atenção Domiciliar, centros especializados, espaços de reabilitação, entre outros.

São dados subnotificados que levam a uma reflexão de que a persistência da violência contra esse grupo reforça a urgência de políticas públicas efetivas, protetivas e afetivas para combater crimes de ódio relacionados à homofobia. Como pode ser observado, o homicídio ocupa o primeiro lugar da causa de morte e o suicídio o segundo, o que traduz a visão deturpada e preconceituosa sobre velhice e homossexualidade, contribuindo para o desencadeamento de sofrimento psíquico^(9,17)^, o que exige um reposicionamento dos profissionais de saúde, na perspectiva da afirmação da identidade sexualmente diversa.

Em 2022, no Brasil, 13% da população mundial eram pessoas idosas, equivalente a 1,1 bilhão de indivíduos^(18)^, sendo 14 milhões de homens idosos com expectativa de vida de 73 anos, sete anos a menos do que as mulheres^(5,19)^, um dado comprobante que o cuidado é dessemelhante e o fragmento da equidade de gênero precisa ser superado. Mundialmente, essa diferença atenua um pouco, mas ainda homens vivem cerca de 4,8 anos a menos. A Pesquisa Nacional de Saúde^(20)^, em 2019, contabilizou 159,2 milhões de habitantes acima de 18 anos de idade; desses, 46,8% são homens e 1,9% autodeclaram-se *gays*. Observa-se que quanto maior a idade, menor é a taxa de autodeclaração da orientação sexual, sugerindo que o público prefere ocultar o dado por questões de insegurança em assumir a homossexualidade.

A ocultação de informações sobre a orientação sexual pode estar relacionada a uma estratégia de autopreservação diante do estigma percebido, que antecipa a discriminação a qual estão sujeitos nos espaços de interação com os profissionais de saúde. No entanto, ao mesmo tempo que protege a identidade deteriorada, a ocultação impede o avanço de ações coletivas do grupo porque o invisibiliza.

Hipóteses apontam para a vertente de que a saúde do homem idoso *gay* não é prioridade em ações coletivas e individuais^(21,22,23)^ devido a falha sociopolítica e disparidades no cumprimento dos princípios da igualdade em relação ao público heterossexual^(23)^.

A pretensão em tela volta-se a uma reflexão direcionada do cenário social, político e de saúde que oferece uma gama de informações sobre o público masculino *gay*, frente aos desafios e obstáculos existentes no envelhecimento e no acesso aos serviços de saúde que muitas vezes os forçam a ocultar sua identidade^(24)^ e, principalmente, disponibiliza um material teórico e norteador para pensar em práticas afirmativas durante atendimentos na saúde interdisciplinar, com o intuito de avançar no cumprimento de políticas que visam acolhimento humanizado das velhices e suas especificidades. Diante da problemática, objetivou-se refletir sobre os cuidados de Enfermagem Afirmativa como apoio à expressão da orientação sexual de homens idosos *gays*.

## MÉTODO

Ensaio teórico-reflexivo com foco no contexto de proteção social e bem-estar do público-alvo, desenvolvido de 2023 a 2025, produto da tese de doutorado do primeiro autor intitulada “Homens mais velhos e idosos cisgêneros e a homoafetividade: da prospecção tecnológica à proposição para a prática de enfermagem e saúde afirmativa”, guiado pela seguinte pergunta: quais são as reflexões teóricas que levam à compreensão dos cuidados de Enfermagem Afirmativa como apoio à expressão da orientação sexual de homens idosos *gays*, com vistas à transição para um envelhecimento saudável?

Tal questão possibilitou o direcionamento do aprofundamento narrativo e do encontro dos atributos do objeto de investigação, por meio da seleção de 47 materiais publicados, sendo: 17 artigos científicos nacionais e 13 internacionais, via *Scielo* e *PubMed/MedLine*, sete materiais governamentais nacionais, uma resolução do Conselho Federal de Enfermagem, dados estatísticos de um observatório, três materiais de dados estatísticos nacionais e dois internacionais, um manual internacional, um livro e um capítulo de livro nacionais encontrados no *Google Acadêmico* e em *sites* oficiais. Empregaram-se os descritores Saúde do Homem, Saúde do Idoso, Enfermagem, Minorias Sexuais e de Gênero e Política Pública. A equipe de pesquisa tem formação na área de Enfermagem, Saúde Coletiva e Gerontologia, além de experiência em pesquisas sobre homossexualidade masculina.

Foram incluídos materiais que abordassem elementos do objeto de interesse como o fenômeno do envelhecimento masculino *gay* e nuances relacionadas a aspectos sociais, políticas públicas, formas de cuidados, relações entre homossexualidade e repercussões em saúde biopsicossocial, materiais sem limite temporal, em português do Brasil, espanhol e inglês (idiomas dos autores). Arquivos corrompidos foram excluídos.

A análise foi realizada por meio da leitura interpretativa baseada no referencial político da Ação Afirmativa^(3)^, guiada por um *checklist* de criação própria contendo itens-chaves como: o texto descreve microfenômenos sociais e políticos relacionados a homens idosos *gays*, mesmo incluindo outros públicos de minorias sexuais? O conteúdo descreve políticas públicas voltadas à saúde masculina *gay* no contexto gerontológico? O material aborda dificuldades de acesso, agravos em saúde e cuidados na perspectiva da ação afirmativa? Os aspectos éticos foram respeitados na perspectiva da garantia da fidedignidade, veracidade e qualidade dos dados produzidos/analisados.

## DESENVOLVIMENTO

### Saúde e Adoecimento na Velhice de Homens *Gays* e Enfermagem Afirmativa Como um Campo Para Garantia de Direitos ao Cuidado

A Enfermagem Afirmativa, fundamentada em princípios do cuidado culturalmente sensível, junto da gerontologia social, propõem práticas que promovem ambientes seguros e fortalecem vínculos terapêuticos, alinhando-se às políticas públicas da saúde do homem *gay*, visando maior adesão assistencial^(7,13,25,26,27)^. Salienta-se que abordagens afirmativas e de suporte social podem ser determinantes para o bem-estar subjetivo e a integração social ao oferecer mecanismos de apoio que mitigam os efeitos deletérios da discriminação de longa duração pela redução do sofrimento psicossocial^(2)^.

Ao longo do século XXI, a ampliação dos espaços institucionais e simbólicos para o debate sobre a população *gay* no campo da saúde tem favorecido maior visibilidade social. Esse movimento, impulsionado por políticas intersetoriais nas áreas da cultura, educação, trabalho, saúde, previdência e justiça, fortaleceu a agenda de enfrentamento à homofobia e consolidou a atenção integral à saúde como eixo estruturante das ações afirmativas^(7)^. Logo, acredita-se na tendência da Enfermagem Afirmativa como propulsora da facilitação da garantia dos direitos e justiça social, mediante reconhecimento e valorização da pessoa socialmente referenciada, tal como da advocacia. Entretanto, mesmo com avanços normativos, a efetivação do cuidado permanece desigual e demanda instrumentalização das práticas profissionais para responder demandas emergentes^(10)^.

No contexto brasileiro, marcos institucionais como o programa *Brasil sem Homofobia* (2004), a 1ª Conferência Nacional LGBT+ (2008) e a incorporação da política LGBT+ pelo Conselho Nacional de Saúde (2011) constituíram avanços importantes na articulação entre direitos sexuais e políticas públicas de saúde, inclusive em diálogo com as políticas de saúde do homem, da pessoa idosa e de saúde mental^(7,13)^. Contudo, a existência desses dispositivos não garante, por si só, a transformação das práticas cotidianas de cuidado, reforçando a necessidade de modelos assistenciais que incorporem a ação afirmativa como eixo estruturante da clínica ampliada.

As experiências discriminatórias, tanto recentes quanto ao longo da vida, preveem maior adoecimento psicológico e pior autopercepção de saúde entre homens idosos *gays* ^(17,28,29)^, o que corrobora a ideia de que políticas inclusivas, quando não acompanhadas de mudanças nas práticas assistenciais, produzem impactos limitados na redução das iniquidades em saúde. Neste âmbito, reflete-se: a conduta, posição e atendimento profissional de enfermagem encontra-se culturalmente competente e congruente para a diversidade sexual e de gênero das pessoas?

O foco prevalente nas abordagens neoliberais de saúde pública que priorizam a responsabilidade pessoal e determinam as práticas sexuais de homens *gays* como risco, aliado ao pouco conhecimento sobre como essas promovem e protegem a saúde de maneira mais abrangente, reflete na pouca atenção dada aos contextos sociais, econômicos e pessoais da vida e às interseções entre contexto e comportamento, uma vez que a homofobia é um fator comprovadamente contribuinte para as desigualdades em saúde e reflete em uma gestão ineficaz da saúde^(30)^, pois se o sistema fomenta preconceito de forma velada, o avanço das atitudes afirmativas fica estacionado, uma vez que o sistema são pessoas.

A Enfermagem Afirmativa, consequentemente, visa respeitar a expressão das pessoas/pacientes, quer seja no âmbito da orientação sexual como na identidade de gênero, recorrendo a recursos que utilizem da linguagem inclusiva, adequação vocal, compreensão de terminologias, do cuidado respeitoso, da criação de ambientes seguros, criativos, da comunicação centrada na pessoa e na sua subjetividade, na evitação de suposições e normatizações compulsórias. Outrossim, na aproximação com a cultura desses homens, das mudanças sistêmicas e dos processos de transições existentes.

Compreender esses processos continuamente e em constante evolução é o primeiro passo para o desenvolvimento de recursos personalizados, que respeitem os contextos sócio-históricos e, ao mesmo tempo, busquem superar as adversidades atuais. Em essência, os efeitos deletérios dos enjeitamentos necessitam ser reconhecidos e abordados simultaneamente para um melhor atendimento em saúde^(31)^, orientado para a construção de relações de confiança e redução das disparidades em saúde.

A vulnerabilidade vivenciada por essa comunidade caracteriza-se pelos estressores minoritários únicos atribuídos a interseccionalidades como idade, capacitismo, etnia, situação profissional^(32)^ e a principal classe de interesse, a homossexualidade, pois apesar de esta última constituir uma dimensão estruturante da sexualidade humana relacionada à forma como os sujeitos vivenciam vínculos afetivo-amorosos e sexuais com pessoas do mesmo gênero^(4,7)^, ainda está longe de se integrar respeitosamente aos direitos humanos fundamentais e à liberdade de expressão igualitária e com segurança perante a lei e a proteção contra práticas opressoras^(1,7,33)^. Isso vai ao encontro de quadros de homofobia internalizada, ao começarem de fato a internalizar a repulsa atrelada à homossexualidade e a construir um autoconceito negativo da própria orientação sexual, necessitando de estratégias de *coping* e autoaceitação.

Vale ressaltar que a sociedade enxerga pessoas idosas desvinculando questões sobre sexualidade a elas e, quando o assunto entra em pauta, surge outro fator excludente que é a crítica destrutiva e perversa sobre comportamento inapropriado na lógica social de que a velhice deve ser isenta de usufruir dos prazeres da vida e de satisfações íntimas. Na sociedade, veiculam-se representações assexuadas na velhice, que se intercruzam a comportamentos sexuais exacerbados e desviantes. Essas ideias fomentam expectativas de que pessoas idosas em geral devam abdicar do desejo, dos prazeres e da intimidade entre os pares. Um cuidado afirmativo considera a necessidade de investir na circulação de ideias e imagens que confrontem esse cenário que nega a sexualidade e impõe controle sobre corpos que envelhecem.

Torna-se central, então, articular o envelhecimento à interseccionalidade entre idade, gênero e orientação sexual, pois estes estão inseridos em processos discriminatórios, reconhecendo as vivências das trajetórias de saúde marcadas por desigualdades cumulativas, tornando a ação afirmativa em saúde em estratégia para a criação de intervenções personalizadas e sensíveis a traumas vivenciados^(2)^.

Com a existência de fragmentos no cumprimento da equidade que levam esse público a enfrentar dessemelhanças estruturais no acesso aos serviços de saúde e em suporte social, condicionadas por estressores acumulados ao longo da vida como, por exemplo, a menor probabilidade de serem casados e terem família constituída^(27)^, amplia-se a disparidade entre a existência formal de direitos e a vivência efetiva de um cuidado acolhedor^(13)^.

Nesse ínterim, há um risco aumentado para o estabelecimento de diagnósticos como depressão e ansiedade em comparação a homens heterossexuais e também transtornos psicológicos graves como consequências do apoio emocional inadequado e do tratamento desigual comunitário, agravados pela discriminação interpessoal^(2)^. Isso delineia oportunidades para fortalecer fatores de proteção como segurança econômica e apoio social, atenuando o risco de adoecimento^(27)^, uma vez que abordagens afirmativas reduzem sintomas psicoemocionais ao promoverem reconhecimento e segurança relacional^(3)^.

Os atravessamentos sociais e institucionais destacam experiências de solidão e invisibilidade social prejudiciais ao processo de envelhecimento^(2,8)^, o que ocasiona a vivência de experiências constrangedoras durante atendimentos em saúde em contextos globais. Essa situação requer dos profissionais de saúde o reconhecimento da realidade com foco na mitigação de atendimentos desproporcionais, ao adotar estratégias que promovam a afirmação da orientação sexual, bem como abordagem psicossocial, cultural e espiritual para um cuidado holístico e compassivo^(32)^, com a possibilidade de suprir engajamentos fragmentados em cuidados preventivos e de longo prazo.

A violência, incluindo suas formas simbólicas tidas como “brincadeiras”, configura-se como um marcador social relevante neste contexto, sustentada por estruturas heteronormativas que valorizam determinados arranjos e desvalorizam orientações sexuais dissidentes ao considerar a heterossexualidade superior, a única normal, natural, correta e válida por seguir um modelo hegemônico dominante^(22,34)^. Os homossexuais são tratados com termos pejorativos como *“tias que fazem [relação sexual] o adolescente”, “viados-Susana-Vieira”, “bichas velhas” e “mariconas caquéticas”*, levando a uma reflexão de que não são seres abjetos nem imorais e sim, cidadãos éticos e morais que precisam, a cada dia, reinventar seu próprio ente, uma vez que a velhice *gay* é tida como rejeitada pela cultura heterossexista^(35)^. Com isso, a Enfermagem Afirmativa volta-se à correção das diferenças estruturais e reconhecimento social^(3)^.

Precisa-se considerar a existência do uso de linguagem rotuladora, atitudes/práticas excruciantes, descorteses, violentas e negligentes (da sociedade e dos cuidadores), e a partir disso assumir uma postura vigilante diante do público a ser assistido. Propomos que um primeiro passo seja a abertura ao diálogo, com escuta sensível daquilo que o grupo espera do cuidado, suas queixas, críticas, sugestões, formação orientada por teorizações sobre gênero, sexualidade e direitos humanos, menor aderência aos modelos focados em queixas clínicas e apoio em modelos holísticos de cuidado que coloquem a pessoa e sua história como eixo/foco do encontro. Face a isso, no contexto brasileiro, a ação afirmativa consolidou-se como instrumento de justiça social ao longo do processo de redemocratização, orientando políticas públicas voltadas à inclusão e à equidade^(3,4)^.

A combinação de cuidados afirmativos estruturais e em saúde mental pode reconhecer a experiência negativa cumulativa e levar ao alcance de melhoria em saúde, na lógica do seu conceito ampliado, buscando sempre uma reflexão por parte dos profissionais em reorganizar seus atendimentos, na atenção básica ou em qualquer outro setor, sempre relembrando de que a população tem o direito de ser atendida livre de qualquer tipo de preconceito e que é proibida qualquer exclusão baseada em idade, orientação sexual e outras classes que se interseccionam^(7,36)^.

Essa ideia de reorientar práticas assistenciais deve-se à frequente redução da população *gay* a marcadores orgânicos específicos que mascaram outras demandas relacionadas ao envelhecimento, subjetividades e relações sociais e que, em algumas realidades, pressupõe-se que a estrutura familiar é apenas no modelo heterossexual e cisgênera, engessando a assistência que não considera, a princípio, a diversidade dos núcleos familiares^(7,21,22,33,37)^.

A falta de articulação entre a dimensão sociocultural do demérito e o cuidado ofertado nos estabelecimentos de saúde compromete a confiança e intensifica o não saber o que fazer e aonde ir quando precisa de ajuda/suporte, refletindo um padrão que ecoa em diferentes contextos internacionais e necessita ser superado^(10,24)^. Soma-se a isso a associação entre envelhecimento, aumento de possíveis problemas de saúde biopsicológica e o levar uma vida de forma precária com incapacidades, fatores esses críticos para manter uma qualidade de vida.

Recomenda-se reflexionar a assistência sustentada na falência do modelo biomédico, ao olhar para a população *gay* e visualizar apenas aspectos clínicos e patológicos relacionados ao aparato sexual de maneira equivocada e negligenciada, e que o entorno social e as interações humanas carecem de implicações nos resultados de saúde na velhice (comunidade, amigos, família, trabalho dentre outros), ao se focar na neutralidade aparente da visão biológica, que incorpore abordagens sensíveis e dialogadas^(3,4)^, facilite o acesso^(33)^ e reconheça que a universalidade formal não elimina barreiras institucionais, pois a mudança geralmente ocorre no processo de autoconscientização ancorado no princípio da igualdade material, da valorização das diferenças, da promoção da cidadania homoafetiva, do fortalecimento das redes de proteção social e do equilíbrio real^(3,4,5,7,13,38)^, rompendo de certa forma, com a dicotomia privilégio-opressão.

As diretrizes nacionais que orientam o cuidado integral, como a obra “Homens *Gays* e Bissexuais: Direitos, Saúde e Participação Social”, os encaminhamentos da 2ª Conferência Nacional LGBT+^(7)^ e as recomendações do “Guia de Cuidados para a Pessoa Idosa”^(13)^, especialmente no que se refere à Avaliação Geriátrica Ampla (AGA), são possíveis recursos de implementação. A AGA, em especial, por se tratar de uma abordagem para além do corpo físico, possibilita um diagnóstico de camadas mais existencialistas, inclusive para aqueles em situação de vulnerabilidade socioeconômica. Pessoas idosas com limitações geográficas também podem usufruir da Telessaúde^(39)^, reduzindo barreiras relacionadas à desinformação e à oferta desigual de serviços^(4,40)^ ao ampliar as possibilidades de acesso ao cuidado afirmativo.

Essas diretrizes, quando articuladas à consulta de enfermagem e ao planejamento terapêutico compartilhado, favorecem práticas de reabilitação funcional, com pactuação de compromissos efetivos entre profissionais, usuários e serviços^(1,41,42)^, pois é um modelo que reconhece singularidades e promove autonomia e participação ativa no cuidado. No plano ético-legal, a Enfermagem Afirmativa encontra respaldo no Código de Ética dos Profissionais de Enfermagem^(43)^ e na Carta dos Direitos dos Usuários da Saúde^(44)^, que asseguram o respeito à dignidade, liberdade de expressão da orientação sexual, oferta de atendimento livre de discriminação e manutenção da esperança.

Dessa forma, o envolvimento da despatologização da homossexualidade nas atividades profissionais, a análise das corporalidades e da diversidade funcional no envelhecimento, bem como a criação de espaços coletivos – presenciais ou virtuais – para compartilhamento de experiências, são estratégias de enfrentamento para a promoção do bem-estar psicossocial^(5,29)^. Tais elementos configuram um cuidado construído de forma coletiva e interdisciplinar alinhados às proposições contemporâneas da enfermagem gerontológica e que podem contribuir para o descongestionamento dos serviços tradicionais de saúde pública de maneira sustentável e inteligente.

Acerca dos pressupostos da saúde coletiva, a enfermagem, juntamente a outras disciplinas como a psicologia, serviço social e medicina, necessita focar em registrar e dar visibilidade às narrativas dos sujeitos, valorizando suas memórias e experiências, ao contrário de impor um único modo de ser ou se relacionar. Deveria inserir todos em debates mais amplos sobre a importância de se atualizar e respeitar o que é plural^(45)^, ao explorar experiências e formas de ser homem sendo homem *gay* e repensar a postura de resistência e singularidade em um contexto social, que muitas vezes impõe padrões que são prejudiciais e excludentes^(46)^, reconhecendo-se que a diversidade sexual extrapola classificações fixas e a sua manifestação volta-se a questões subjetivas individuais.

O estudo da diversidade da sexualidade precisa ser plenamente integrado à gerontologia, pois frisar os importantes papéis da afirmação e da autonomia contribui para o reconhecimento dos direitos humanos como essenciais na promoção da qualidade de vida sexual^(29)^. Por isso, a ideia cuidativa abordada neste ensaio configura-se como um campo emergente e estratégico que articula a produção do cuidado com diferentes campos/disciplinas que se interligam na prática, pois ao reconhecer a singularidade das trajetórias de vida, das masculinidades e das experiências de envelhecimento, profissionais contribuirão para a redução de iniquidades, o fortalecimento da cidadania e a construção de itinerários terapêuticos sensíveis não somente às dimensões clínicas.

## POTENCIALIDADES E IMPLICAÇÕES PARA A PRÁTICA DE ENFERMAGEM

Esse é um fenômeno ainda pouco explorado pela enfermagem e uma produção de dados sobre um grupo historicamente excluído pode estimular a execução estratégica de modelos em espaços interculturais^(3)^, capazes de fortalecer o reconhecimento da Enfermagem Afirmativa como um campo clínico-social e profissional equânime que implementa intervenções focadas na superação de traumas por meio da abordagem de questões subjacentes que ajudam a cultivar os relacionamentos de apoio, sociais, de resiliência e de identidade positiva, pois são preditores significativos do bem-estar e de um envelhecimento bem-sucedido^(47)^.

Sabe-se que compreender a configuração de recursos e riscos por faixa etária e orientação sexual é importante para o desenvolvimento de iniciativas em saúde adaptadas ao reconhecimento do papel sistêmico da discriminação, ao mesmo tempo que apoia esforços individuais no gerenciamento ativo de saúde, levando em consideração a heterogeneidade na compreensão da sexualidade e suas dimensões na velhice.

Tais elementos podem ser desenvolvidos durante a oferta de atendimentos afirmativos, e o que se constrói ao longo das reflexões é a ideia de que o campo interdisciplinar que atua na área da saúde precisa ter um olhar prático e sensível para superar sistemas interligados de opressão por meio de conexões sociais significativas, tais como fomentar relacionamentos de autenticidade e profundidade com o propósito de cultivar um acompanhamento longitudinal e antiestigmatizante^(2)^ focado na promoção do apoio à expressão da orientação sexual.

Para auxiliar estrategicamente a ideia de melhorias nos atendimentos, torna-se relevante realizar capacitações culturalmente dos profissionais de saúde por meio de treinamentos unificados quanto à duração, conteúdo e metodologia, principalmente quando termos/terminologias-chaves, sexualidade, anamnese sexual (disfunções), saúde específica de homens idosos *gays* e disparidades em saúde são abordados. Isso tem resultado em melhora do conhecimento em curto prazo de tal público frente às necessidades da homossexualidade de homens idosos, sempre os incluindo nos processos formativos de educação permanente.

O avanço do conhecimento da diversidade da orientação sexual e etária e a estruturação de um novo cuidar com perspectivas de abordagens ampliadas na contratação de enfermeiros homens *gays*, na intenção de compor equipes que poderão desenvolver a autonomia do sujeito e refletir em aproximação e troca de ideias mais efetiva, são pontos a se pensar dentro das instituições, como também o estímulo para ofertar disciplinas específicas na graduação e pós-graduação em uma perspectiva curricular mais diversa, fluida e próxima do contexto atual, uma vez que a formação é ainda focada na heterossexualidade.

## CONSIDERAÇÕES FINAIS

Em que pese o registro dos avanços nas políticas afirmativas e nos documentos orientadores existentes no contexto brasileiro, os obstáculos para a efetividade dos cuidados em Enfermagem Afirmativa como apoio à expressão da orientação sexual de homens idosos *gays* são derivados da conjunção de negligências, carências e ausências no campo prático do cuidado. A ausência de abordagens inclusivas nos currículos, o silêncio sobre a sexualidade e os tabus relacionados ao corpo de pessoas idosas justificam a ideia de criar uma tendência cuidativa integrada à política de ação afirmativa como forma de reduzir o preconceito social e institucional e aumentar a adesão desses homens aos serviços de saúde.

Práticas congruentes com as necessidades desses homens precisam ser configuradas para zelar por sua dignidade em todos os ciclos do desenvolvimento, preservando-os de qualquer tratamento desumano, violento, aterrorizante, vexatório ou constrangedor na medida em que eles envelhecem.

Espera-se que, futuramente, seja possível uma sistematização técnica e científica mais estruturada e institucionalizada para que o julgamento clínico de problemas que afloram respostas humanas seja mais bem executado e possibilite transições saudáveis desses personagens sociais com maestria. Afinal, cuidar da saúde do homem idoso *gay* é possível com base no entendimento de suas demandas e não somente no cumprimento dos deveres e sim, da compreensão coletiva da importância na oferta de proteção.

Enfermeiros necessitam desenvolver estudos voltados à compreensão da saúde de homens idosos *gays*, pois este é um fato que limitou a discussão desta produção de maneira mais direcionada, já que a maioria dos estudos é generalizada e envolve toda a população de minorias sexuais, de gênero, de identidade e de expressão de gênero. Portanto, é necessário delimitar o público que deseja explorar, pois as particularidades tendem a ser diferentes, como também realizar investigações que mensurem variáveis de saúde mental e desafios sociais relacionados à homossexualidade masculina, especificamente, pois são temas que se associam.

## DISPONIBILIDADE DE DADOS

Todo o conjunto de dados que dá suporte aos resultados deste estudo foi publicado no próprio artigo.

# Translation: Reflexión conceptual sobre la Enfermería Afirmativa como campo profesional para el cuidado de hombres homosexuales mayores

**DOI:** 10.1590/1980-220X-REEUSP-2025-0517es

**Authors:** Vinicius de Oliveira Muniz, Pricila Oliveira de Araújo, Evanilda Souza de Santana Carvalho, Rosalina Aparecida Partezani Rodrigues, Anderson Reis de Sousa

**Affiliations:** 1 Universidade Federal da Bahia, Escola de Enfermagem, Salvador, BA, Brasil.; 2 Universidade Estadual de Feira de Santana, Feira de Santana, BA, Brasil.; 3 Universidade de São Paulo, Escola de Enfermagem de Ribeirão Preto, Ribeirão Preto, SP, Brasil.

**Keywords:** Salud del Hombre, Salud del Anciano, Enfermería, Minorías Sexuales y de Género, Política Pública

## Abstract

**Objetivo:**

Reflexionar sobre la atención de Enfermería Afirmativa como apoyo a la expresión de la orientación sexual en hombres mayores homosexuales.

**Método:**

Un estudio teórico, desarrollado entre 2023 y 2025, que incluye artículos, materiales gubernamentales, observatorios, un manual, un libro/capítulo de libro y una resolución. El análisis se realizó a través de la lectura interpretativa, basada en el marco de la Acción Afirmativa, guiada por *lista de verificación*.

**Desarrollo:**

La heteronormatividad refleja un escenario problemático que estos hombres experimentan en todo el mundo, rodeados de homofobia, violencia simbólica, marginación de los servicios de salud debido a experiencias vergonzosas, autosegregación, doble estigmatización, exclusión social e invisibilidad, lo que contribuye a un *Coming Out* dañado. La Enfermería Afirmativa se basa en principios de atención afectiva, inclusiva y culturalmente sensible. Junto con la gerontología social y de las políticas públicas, proponen prácticas que reconocen identidades, promueven entornos seguros y fortalecen vínculos terapéuticos.

**Consideraciones finales:**

Crear una tendencia de cuidados integrada a las políticas de acción afirmativa es una forma de reducir los prejuicios y aumentar la adherencia a los servicios de salud, una práctica que debe configurarse para salvaguardar la dignidad de los hombres mayores homosexuales, protegiéndolo de cualquier trato inhumano y/o vergonzoso.

## INTRODUCCIÓN

Los hombres *gays* de edad avanzada han sido presionados socialmente a pensar la sexualidad como enfermedad mental, desviación de la salud o inmoralidad, lo que ha llevado a la mayoría de ellos a vivir su homosexualidad creyendo que la heterosexualidad sería algo superior y la única forma de expresar la orientación sexual^(1)^. A esto se suman las experiencias de discriminación continua, la crisis del VIH/sida y los retos para afrontar las complejidades del envejecimiento^(2)^, lo que contribuye a la necesidad de fortalecer la atención en salud, desde la perspectiva de la Enfermería Afirmativa, basada en iniciativas de suspensión de métodos de exclusión social que validen la diversidad y celebren las identidades al reconocerlas como puntos fuertes^(3,4)^.

El enfoque de la “Enfermería Afirmativa” argumenta a través de un diálogo cercano a los constructos de la atención afirmativa^(4)^, la política afirmativa^(5)^ y la acción afirmativa^(3)^, debido a configuraciones orientadas a estrategias de lucha contra la desigualdad por orientación sexual, utilizando en la práctica clínica instrumentos de reparación histórica, antidiscriminatorios y/o de compensación de desventajas derivadas de las estructuras, comportamientos y actitudes^(3,4)^. Como práctica, exige un desarrollo sistemático y científico desde una perspectiva política y ética, ya que desafía las normas cisheteronormativas prevalentes en los servicios de salud en general. Así, se ocupa de superar la falta de orientación teórica de la práctica, la escasez y el vaciamiento de sentido y propósito profesional en la atención a las demandas y necesidades singulares de los sujetos.

La doble discriminación por edad y orientación sexual puede ejemplificar un escenario en el que se produce la interseccionalidad y en el que se cruzan múltiples identidades marginadas, lo que sustancia agravios y vulnerabilidades sociales, en la medida en que se amplifican las desigualdades y las opresiones^(6)^. Factores adicionales como el color de la piel y la condición socioeconómica tienden a potenciar estas desigualdades, con la capacidad de revelar, tal vez, que las personas mayores *gays* negras presentan indicadores críticos de acceso a la salud dentro de este colectivo, lo que contribuye al sentimiento de culpa, tristeza, humillación y carga social^(7,8,9)^.

Además, se observa que la sociedad fomenta el miedo a la vejez al asociarla con la enfermedad, exalta la juventud y margina a las personas mayores, además de negarles el derecho a ejercer su sexualidad, lo que tiende a que las clases interseccionales estén presentes en la vida cotidiana de estas personas. Cuando se trata de hombres mayores *gais* que acuden a los servicios de salud, estos ocultan su orientación sexual, lo que se caracteriza como un *Coming Out* perjudicial^(10)^, es decir, permanecer “en el armario” como estrategia para hacer frente al prejuicio, con el fin de proteger la identidad (preservar el yo), pero que, en consecuencia, contribuye a una baja autoestima, al desapego social, a la represión y al aislamiento. Esta inseguridad puede implicar transiciones insalubres durante los ciclos vitales y el envejecimiento.

Los estudios sobre los cuidados dirigidos a los hombres mayores han explorado poco la salud de los hombres *gays*, tanto en el campo específico de la enfermería como en el interdisciplinario de la geriatría y la gerontología, demostrando la unidireccionalidad y el reduccionismo de solo algunas de las representaciones LGBTQIAPN+^(11)^. Esta laguna implica la vivencia saludable de la sexualidad y la expresión segura de la homosexualidad, lo que también se percibe en las directrices de la Política Nacional de Salud de la Persona Mayor^(12)^ y en la Guía de Cuidados para la Persona Mayor^(13)^, ambas publicadas en Brasil, ya que abordan brevemente la cuestión del envejecimiento homoafectivo, sin señalar caminos para una planificación/gestión sistematizada de las demandas de cuidados.

Esta invisibilidad se refleja en los estudios sobre violencia. Aunque Estados Unidos (EE. UU.) ha registrado que el 17,2 % de las 14 243 víctimas de delitos de odio fueron motivados por la orientación sexual, entre los que se incluyen homicidios, agresiones y otros tipos de violencia, no hay datos específicos sobre hombres mayores *gays* ^(14)^. En Brasil, se produjeron 165 homicidios de hombres *gais* en 2024, sin especificar el grupo de edad. En 2019, el 3,9 % de las muertes de personas LGBT+ fueron de hombres mayores *gais*, de entre 68 y 70 años, en las regiones del norte y noreste de Brasil. Ceará, São Paulo y Pernambuco son los estados con el mayor número de víctimas^(15)^.

A pesar de constituir una proporción significativa de los eventos violentos, no hay un número agregado mundial confiable disponible que incluya la orientación sexual en los sistemas de datos de defunción en muchos países, ni una recolección oficial de datos debido a la criminalización de las relaciones homoafectivas, como, por ejemplo, en los continentes asiático y africano, lo que contribuye a la fragmentación de las notificaciones y a que no se refuercen las estrategias de sensibilización a largo plazo^(16)^, ya que el propio sistema político es discriminatorio en estas regiones y despierta la atención dirigida a la atención sanitaria prestada en la atención primaria, hospitales, ambulatorios, atención domiciliaria, centros especializados, espacios de rehabilitación, entre otros.

Se trata de datos subnotificados que llevan a reflexionar sobre cómo la persistencia de la violencia contra este grupo refuerza la urgencia de políticas públicas eficaces, protectoras y afectivas para combatir los delitos de odio relacionados con la homofobia. Como se puede observar, el homicidio ocupa el primer lugar entre las causas de muerte y el suicidio el segundo, lo que refleja la visión distorsionada y prejuiciosa sobre la vejez y la homosexualidad, contribuyendo al desencadenamiento del sufrimiento psíquico^(9,17)^, lo que exige un reposicionamiento de los profesionales de la salud, desde la perspectiva de la afirmación de la identidad sexualmente diversa.

En 2022, en Brasil, el 13 % de la población mundial eran personas mayores, lo que equivale a 1100 millones de individuos^(18)^, siendo 14 millones de hombres mayores con una esperanza de vida de 73 años, siete años menos que las mujeres^(5,19)^, un dato que demuestra que la atención es desigual y que es necesario superar la brecha de equidad de género. A nivel mundial, esta diferencia se atenúa un poco, pero los hombres siguen viviendo unos 4.8 años menos. La Encuesta Nacional de Salud^(20)^ de 2019 contabilizó 159.2 millones de habitantes mayores de 18 años; de ellos, el 46.8 % son hombres y el 1.9 % se autodeclaran *gais*. Se observa que cuanto mayor es la edad, menor es la tasa de autodeclaración de la orientación sexual, lo que sugiere que el público prefiere ocultar este dato por cuestiones de inseguridad a la hora de asumir la homosexualidad.

El ocultamiento de información sobre la orientación sexual puede estar relacionado con una estrategia de autoconservación ante el estigma percibido, que anticipa la discriminación a la que están sujetos en los espacios de interacción con los profesionales de la salud. Sin embargo, al mismo tiempo que protege la identidad deteriorada, el ocultamiento impide el avance de las acciones colectivas del grupo porque lo invisibiliza.

Las hipótesis apuntan a que la salud del hombre gay de edad avanzada no es una prioridad en las acciones colectivas e individuales^(21,22,23)^ debido al fracaso sociopolítico y a las disparidades en el cumplimiento de los principios de igualdad en relación con el público heterosexual^(23)^.

La pretensión en cuestión se centra en una reflexión dirigida al escenario social, político y en salud que ofrece una amplia gama de información sobre el público masculino gay, frente a los retos y obstáculos existentes en el envejecimiento y el acceso a los servicios de salud, que a menudo les obligan a ocultar su identidad^(24)^ y, sobre todo, ofrece material teórico y orientativo para pensar en prácticas afirmativas durante la atención sanitaria interdisciplinaria, con el fin de avanzar en el cumplimiento de políticas que apuestan por una acogida humanizada de la vejez y sus especificidades. Ante esta problemática, se planteó reflexionar sobre los cuidados de Enfermería Afirmativa como apoyo a la expresión de la orientación sexual de los hombres mayores gays.

## MÉTODO

Ensayo teórico-reflexivo centrado en el contexto de la protección social y el bienestar del público objetivo, desarrollado entre 2023 y 2025, producto de la tesis doctoral del primer autor titulada “Hombres mayores y ancianos cisgénero y la homoafectividad: de la prospección tecnológica a la propuesta para la práctica de la enfermería y la salud afirmativa”, guiado por la siguiente pregunta: ¿cuáles son las reflexiones teóricas que llevan a la comprensión de los cuidados de Enfermería Afirmativa como apoyo a la expresión de la orientación sexual de los hombres *gais* de edad avanzada, con vistas a la transición hacia un envejecimiento saludable?

Esta pregunta permitió orientar la profundización narrativa y el encuentro de los atributos del objeto de investigación, mediante la selección de 47 materiales publicados, siendo: 17 artículos científicos nacionales y 13 internacionales, a través de *Scielo* y *PubMed/MedLine,* siete materiales gubernamentales nacionales, una resolución del Consejo Federal de Enfermería, datos estadísticos de un observatorio, tres materiales de datos estadísticos nacionales y dos internacionales, un manual internacional, un libro y un capítulo de un libro nacional encontrados en *Google Académico* y en *sitios web* oficiales. Se utilizaron los descriptores Salud del Hombre, Salud del Anciano, Enfermería, Minorías Sexuales y de Género y Política Pública. El equipo de investigación tiene formación en el área de Enfermería, Salud Colectiva y Gerontología, además de experiencia en investigaciones sobre homosexualidad masculina.

Se incluyeron materiales que abordaban elementos del objeto de interés, como el fenómeno del envejecimiento masculino gay y matices relacionados con aspectos sociales, políticas públicas, formas de cuidado, relaciones entre la homosexualidad y repercusiones en la salud biopsicosocial, materiales sin límite temporal, en portugués de Brasil, español e inglés (idiomas de los autores). Se excluyeron los archivos dañados.

El análisis se realizó mediante una lectura interpretativa basada en el marco político de la Acción Afirmativa^(3)^, guiada por una lista de verificación de creación propia que contenía elementos clave como: ¿el texto describe microfenómenos sociales y políticos relacionados con hombres adultos mayores gay*s*, incluso incluyendo otros públicos de minorías sexuales? ¿El contenido describe políticas públicas dirigidas a la salud masculina *gay* en el contexto gerontológico? ¿El material aborda las dificultades de acceso, los problemas en salud y los cuidados desde la perspectiva de la acción afirmativa? Se respetaron los aspectos éticos desde la perspectiva de la garantía de la fiabilidad, veracidad y calidad de los datos producidos/analizados.

## DESARROLLO

### alud y Enfermedad en la Vejez de Los Hombres Gays y la Enfermería Afirmativa Como Campo Para Garantizar Los Derechos a la Atención

La Enfermería Afirmativa, basada en principios de cuidado culturalmente sensible, junto con la gerontología social, propone prácticas que promueven entornos seguros y fortalecen los vínculos terapéuticos, alineándose con las políticas públicas de salud del hombre gay, con el objetivo de lograr una mayor adhesión a la asistencia^(7,13,25,26,27)^. Cabe destacar que los enfoques afirmativos y de apoyo social pueden ser determinantes para el bienestar subjetivo y la integración social, al ofrecer mecanismos de apoyo que mitigan los efectos nocivos de la discriminación a largo plazo mediante la reducción del sufrimiento psicosocial^(2)^.

A lo largo del siglo XXI, la ampliación de los espacios institucionales y simbólicos para el debate sobre la población gay en el ámbito de la salud ha favorecido una mayor visibilidad social. Este movimiento, impulsado por políticas intersectoriales en las áreas de cultura, educación, trabajo, salud, previsión social y justicia, ha fortalecido la agenda de lucha contra la homofobia y ha consolidado la atención integral a la salud como eje estructurante de las acciones afirmativas^(7)^. Por lo tanto, se cree en la tendencia de la Enfermería Afirmativa como impulsora de la facilitación de la garantía de los derechos y la justicia social, mediante el reconocimiento y la valorización de la persona socialmente referenciada, al igual que la abogacía. Sin embargo, incluso con los avances normativos, la efectividad de la atención sigue siendo desigual y exige la instrumentalización de las prácticas profesionales para responder a las demandas emergentes^(10)^.

En el contexto brasileño, hitos institucionales como el programa Brasil sin Homofobia (2004), la 1.ª Conferencia Nacional LGBT+ (2008) y la incorporación de la política LGBT+ por parte del Consejo Nacional de Salud (2011) constituyeron avances importantes en la articulación entre los derechos sexuales y las políticas públicas de salud, incluso en diálogo con las políticas de salud del hombre, de la persona mayor y de salud mental^(7,13)^. Sin embargo, la existencia de estos dispositivos no garantiza, por sí sola, la transformación de las prácticas cotidianas de atención, lo que refuerza la necesidad de modelos asistenciales que incorporen la acción afirmativa como eje estructurante de la clínica ampliada.

Las experiencias discriminatorias, tanto recientes como a lo largo de la vida, predicen un mayor malestar psicológico y una peor autopercepción de la salud entre los hombres mayores *gays* ^(17,28,29)^, lo que corrobora la idea de que las políticas inclusivas, cuando no van acompañadas de cambios en las prácticas asistenciales, producen impactos limitados en la reducción de las desigualdades en salud. En este contexto, cabe reflexionar: ¿la conducta, la posición y la atención profesional de la enfermería son culturalmente competentes y congruentes con la diversidad sexual y de género de las personas?

El enfoque predominante en los enfoques neoliberales de salud pública que priorizan la responsabilidad personal y determinan las prácticas sexuales de los hombres *gais* como un riesgo, junto con el escaso conocimiento sobre cómo estas promueven y protegen la salud de manera más amplia, se refleja en la poca atención que se presta a los contextos sociales, económicos y personales de la vida y a las intersecciones entre el contexto y el comportamiento, ya que la homofobia es un factor que contribuye de manera comprobada a las desigualdades en salud y se refleja en una gestión ineficaz de la salud^(30)^, pues si el sistema fomenta el prejuicio de manera velada, el avance de las actitudes afirmativas se estanca, ya que el sistema son las personas.

La Enfermería Afirmativa, por consiguiente, tiene como objetivo respetar la expresión de las personas/pacientes, ya sea en el ámbito de la orientación sexual o de la identidad de género, recurriendo a recursos que utilicen un lenguaje inclusivo, una adecuación vocal, la comprensión de la terminología, el cuidado respetuoso, la creación de entornos seguros y creativos, la comunicación centrada en la persona y su subjetividad, evitando suposiciones y normativizaciones compulsivas. Además, en el acercamiento a la cultura de estos hombres, a los cambios sistémicos y a los procesos de transición existentes.

Comprender estos procesos de forma continua y en constante evolución es el primer paso para el desarrollo de recursos personalizados, que respeten los contextos sociohistóricos y, al mismo tiempo, busquen superar las adversidades actuales. En esencia, los efectos nocivos del rechazo deben reconocerse y abordarse simultáneamente para mejorar la atención en salud^(31)^, orientada a la construcción de relaciones de confianza y a la reducción de las disparidades en salud.

La vulnerabilidad que experimenta esta comunidad se caracteriza por factores de estrés minoritarios únicos atribuidos a interseccionalidades como la edad, el capacitismo, la etnia, la situación profesional^(32)^ y la principal clase de interés, la homosexualidad, ya que, a pesar de que esta última constituye una dimensión estructurante de la sexualidad humana relacionada con la forma en que los sujetos experimentan los vínculos afectivos, amorosos y sexuales con personas del mismo género^(4,7)^, aún está lejos de integrarse respetuosamente en los derechos humanos fundamentales y en la libertad de expresión igualitaria y con seguridad ante la ley y la protección contra prácticas opresivas^(1,7,33)^. Esto se ajusta a los cuadros de homofobia internalizada, al comenzar de hecho a internalizar el rechazo vinculado a la homosexualidad y a construir un concepto negativo de la propia orientación sexual, lo que requiere estrategias de afrontamiento y autoaceptación.

Cabe destacar que la sociedad ve a las personas mayores desvinculando las cuestiones sobre sexualidad de ellas y, cuando el tema sale a colación, surge otro factor excluyente que es la crítica destructiva y perversa sobre el comportamiento inapropiado en la lógica social de que la vejez debe estar exenta de disfrutar de los placeres de la vida y de las satisfacciones íntimas. En la sociedad se transmiten representaciones asexuadas de la vejez, que se entrecruzan con comportamientos sexuales exacerbados y desviados. Estas ideas fomentan la expectativa de que las personas mayores en general deben renunciar al deseo, los placeres y la intimidad entre parejas. Un cuidado afirmativo considera la necesidad de invertir en la circulación de ideas e imágenes que se enfrenten a este escenario que niega la sexualidad e impone el control sobre los cuerpos que envejecen.

Por lo tanto, resulta fundamental articular el envejecimiento con la interseccionalidad entre edad, género y orientación sexual, ya que estos están insertos en procesos discriminatorios, reconociendo las experiencias de trayectorias de salud marcadas por desigualdades acumulativas, lo que convierte la acción afirmativa en salud en una estrategia para la creación de intervenciones personalizadas y sensibles a los traumas vividos^(2)^.

La existencia de fragmentos en el cumplimiento de la equidad lleva a este público a enfrentarse a desigualdades estructurales en el acceso a los servicios de salud y al apoyo social, condicionadas por factores estresantes acumulados a lo largo de la vida, como, por ejemplo, la menor probabilidad de estar casados y tener una familia constituida^(27)^, lo que amplía la disparidad entre la existencia formal de derechos y la experiencia efectiva de una atención acogedora^(13)^.

Mientras tanto, existe un mayor riesgo de que se establezcan diagnósticos como depresión y ansiedad en comparación con los hombres heterosexuales, así como trastornos psicológicos graves como consecuencia del apoyo emocional inadecuado y el trato desigual de la comunidad, agravados por la discriminación interpersonal^(2)^. Esto plantea oportunidades para fortalecer factores de protección como la seguridad económica y el apoyo social, mitigando el riesgo de enfermedad^(27)^, ya que los enfoques afirmativos reducen los síntomas psicoemocionales al promover el reconocimiento y la seguridad relacional^(3)^.

Las intersecciones sociales e institucionales destacan experiencias de soledad e invisibilidad social perjudiciales para el proceso de envejecimiento^(2,8)^, lo que provoca experiencias incómodas durante la atención en salud en contextos globales. Esta situación requiere que los profesionales de la salud reconozcan la realidad con un enfoque en la mitigación de la atención desproporcionada, adoptando estrategias que promuevan la afirmación de la orientación sexual, así como un enfoque psicosocial, cultural y espiritual para una atención holística y compasiva^(32)^, con la posibilidad de suplir compromisos fragmentados en la atención preventiva y a largo plazo.

La violencia, incluidas sus formas simbólicas consideradas “bromas”, se configura como un marcador social relevante en este contexto, sustentado por estructuras heteronormativas que valoran determinados arreglos y desvalorizan las orientaciones sexuales disidentes al considerar la heterosexualidad superior, la única normal, natural, correcta y válida por seguir un modelo hegemónico dominante^(22,34)^. A los homosexuales se les trata con términos peyorativos como “tías que hacen [relaciones sexuales] *con adolescentes”, “maricas-Susana-Vieira”, “maricas viejos” y “maricas caquécticos”*, lo que lleva a reflexionar que no son seres abyectos ni inmorales, sino ciudadanos éticos y morales que necesitan cada día, reinventar su propio ser, ya que la vejez *gay* es rechazada por la cultura heterosexista^(35)^. Con ello, la Enfermería Afirmativa se centra en la corrección de las diferencias estructurales y el reconocimiento social^(3)^.

Es necesario considerar la existencia del uso de lenguaje etiquetador, actitudes/prácticas crueles, descortesías, violencia y negligencia (por parte de la sociedad y de los cuidadores) y, a partir de ahí, adoptar una postura vigilante ante el público al que se atiende. Proponemos que un primer paso sea la apertura al diálogo, con una escucha sensible de lo que el grupo espera de la atención, sus quejas, críticas, sugerencias, formación orientada por teorías sobre género, sexualidad y derechos humanos, menor adhesión a los modelos centrados en las quejas clínicas y apoyo a modelos holísticos de atención que sitúen a la persona y su historia como eje/foco del encuentro. Ante esto, en el contexto brasileño, la acción afirmativa se consolidó como instrumento de justicia social a lo largo del proceso de redemocratización, orientando las políticas públicas hacia la inclusión y la equidad^(3,4)^.

La combinación de cuidados afirmativos estructurales y en salud puede reconocer la experiencia negativa acumulativa y conducir a la mejora de la salud, en la lógica de su concepto ampliado, buscando siempre una reflexión por parte de los profesionales para reorganizar sus atenciones, en la atención primaria o en cualquier otro sector, recordando siempre que la población tiene derecho a ser atendida libre de cualquier tipo de prejuicio y que está prohibida cualquier exclusión basada en la edad, la orientación sexual y otras clases que se entrecruzan^(7,36)^.

Esta idea de reorientar las prácticas asistenciales se debe a la frecuente reducción de la población gay a marcadores orgánicos específicos que enmascaran otras demandas relacionadas con el envejecimiento, las subjetividades y las relaciones sociales y que, en algunas realidades, se supone que la estructura familiar es solo en el modelo heterosexual y cisgénero, lo que limita la asistencia que no considera, en principio, la diversidad de los núcleos familiares^(7,21,22,33,37)^.

La falta de articulación entre la dimensión sociocultural del desmérito y la atención ofrecida en los establecimientos de salud compromete la confianza e intensifica el no saber qué hacer y adónde acudir cuando se necesita ayuda/apoyo, lo que refleja un patrón que se repite en diferentes contextos internacionales y que es necesario superar^(10,24)^. A esto se suma la asociación entre el envejecimiento, el aumento de posibles problemas de salud biopsicológica y el hecho de llevar una vida precaria con discapacidades, factores críticos para mantener la calidad de vida.

Se recomienda reflexionar sobre la asistencia basada en el fracaso del modelo biomédico, al observar a la población gay y visualizar solo aspectos clínicos y patológicos relacionados con el aparato sexual de manera errónea y negligente, y que el entorno social y las interacciones humanas carecen de implicaciones en los resultados de salud en la vejez (comunidad, amigos, familia, trabajo, entre otros), al centrarse en la aparente neutralidad de la visión biológica, que incorpore enfoques sensibles y dialogados^(3,4)^, facilite el acceso^(33)^ y reconozca que la universalidad formal no elimina las barreras institucionales, ya que el cambio suele producirse en el proceso de auto concienciación basado en el principio de la igualdad material, la valoración de las diferencias, la promoción de la ciudadanía homoafectiva, el fortalecimiento de las redes de protección social y el equilibrio real^(3,4,5,7,13,38)^, rompiendo en cierta medida con la dicotomía privilegio-opresión.

Las directrices nacionales que orientan la atención integral, como la obra “Hombres *gais* y bisexuales: derechos, salud y participación social”, las recomendaciones de la II Conferencia Nacional LGBT+^(7)^ y las recomendaciones de la “Guía de cuidados para la persona mayor”^(13)^, especialmente en lo que se refiere a la Evaluación Geriátrica Amplia (Avaliação Geriátrica Ampla -AGA en portugués), son posibles recursos de implementación. La EGA, en particular, al tratarse de un enfoque que va más allá del cuerpo físico, permite un diagnóstico de capas más existencialistas, incluso para aquellos en situación de vulnerabilidad socioeconómica. Las personas mayores con limitaciones geográficas también pueden beneficiarse de la telesalud^(39)^, lo que reduce las barreras relacionadas con la desinformación y la oferta desigual de servicios^(4,40)^ al ampliar las posibilidades de acceso a la atención afirmativa.

Estas directrices, cuando se articulan con la consulta de enfermería y la planificación terapéutica compartida, favorecen las prácticas de rehabilitación funcional, con el acuerdo de compromisos efectivos entre profesionales, usuarios y servicios^(1,41,42)^, ya que es un modelo que reconoce las singularidades y promueve la autonomía y la participación activa en la atención. En el plano ético-legal, la Enfermería Afirmativa encuentra respaldo en el Código de Ética de los Profesionales de Enfermería^(43)^ y en la Carta de Derechos de los Usuarios de la Salud^(44)^, que garantizan el respeto a la dignidad, la libertad de expresión de la orientación sexual, la oferta de atención libre de discriminación y el mantenimiento de la esperanza.

De este modo, la implicación de la despatologización de la homosexualidad en las actividades profesionales, el análisis de las corporalidades y la diversidad funcional en el envejecimiento, así como la creación de espacios colectivos —presenciales o virtuales— para compartir experiencias, son estrategias de afrontamiento para la promoción del bienestar psicosocial^(5,29)^. Estos elementos configuran una atención construida de forma colectiva e interdisciplinaria, alineada con las propuestas contemporáneas de la enfermería gerontológica, y que pueden contribuir a descongestionar los servicios tradicionales de salud pública de manera sostenible e inteligente.

En cuanto a los supuestos de la salud colectiva, la enfermería, junto con otras disciplinas como la psicología, el servicio social y la medicina, debe centrarse en registrar y dar visibilidad a las narrativas de los sujetos, valorando sus recuerdos y experiencias, en lugar de imponer una única forma de ser o de relacionarse. Debería incluir a todos en debates más amplios sobre la importancia de actualizarse y respetar lo que es plural^(45)^, al explorar experiencias y formas de ser hombre siendo hombre *gay* y replantearse la postura de resistencia y singularidad en un contexto social que a menudo impone patrones perjudiciales y excluyentes^(46)^, reconociendo que la diversidad sexual trasciende las clasificaciones fijas y que su manifestación se centra en cuestiones subjetivas individuales.

El estudio de la diversidad de la sexualidad debe integrarse plenamente en la gerontología, ya que destacar los importantes papeles de la afirmación y la autonomía contribuye al reconocimiento de los derechos humanos como esenciales para la promoción de la calidad de vida sexual^(29)^. Por lo tanto, la idea relacionada al cuidado abordada en este ensayo se configura como un campo emergente y estratégico que articula la producción del cuidado con diferentes campos/disciplinas que se interrelacionan en la práctica, ya que al reconocer la singularidad de las trayectorias de vida, las masculinidades y las experiencias del envejecimiento, los profesionales contribuirán a la reducción de las desigualdades, al fortalecimiento de la ciudadanía y a la construcción de itinerarios terapéuticos sensibles no solo a las dimensiones clínicas.

## POTENCIALIDADES E IMPLICACIONES PARA LA PRÁCTICA DE LA ENFERMERÍA

Este es un fenómeno aún poco explorado por la enfermería, y la producción de datos sobre un grupo históricamente excluido puede estimular la ejecución estratégica de modelos en espacios interculturales^(3)^, capaces de fortalecer el reconocimiento de la Enfermería Afirmativa como un campo clínico-social y profesional equitativo que implementa intervenciones enfocadas en la superación de traumas mediante el abordaje de cuestiones subyacentes que ayudan a cultivar las relaciones de apoyo, sociales, de resiliencia y de identidad positiva, ya que son predictores significativos del bienestar y de un envejecimiento exitoso^(47)^.

Se sabe que comprender la configuración de los recursos y los riesgos por grupo de edad y orientación sexual es importante para el desarrollo de iniciativas de salud adaptadas al reconocimiento del papel sistémico de la discriminación, al tiempo que apoya los esfuerzos individuales en la gestión activa de la salud, teniendo en cuenta la heterogeneidad en la comprensión de la sexualidad y sus dimensiones en la vejez.

Estos elementos pueden desarrollarse durante la prestación de servicios afirmativos, y lo que se construye a lo largo de las reflexiones es la idea de que el campo interdisciplinario que actúa en el área de la salud debe tener una visión práctica y sensible para superar los sistemas interconectados de opresión a través de conexiones sociales significativas, como fomentar relaciones auténticas y profundas con el propósito de cultivar un acompañamiento longitudinal y anti estigmatizante^(2)^ centrado en promover el apoyo a la expresión de la orientación sexual.

Para apoyar estratégicamente la idea de mejorar la atención, es importante capacitar culturalmente a los profesionales de la salud mediante cursos de formación unificados en cuanto a duración, contenido y metodología, especialmente cuando se abordan términos/terminología clave, sexualidad, anamnesis sexual (disfunciones), salud específica de hombres mayores *gays* y disparidades en salud. Esto ha dado lugar a una mejora a corto plazo del conocimiento de este público sobre las necesidades de la homosexualidad de los hombres mayores, incluyéndolos siempre en los procesos formativos de educación permanente.

El avance del conocimiento sobre la diversidad de la orientación sexual y la edad y la estructuración de una nueva atención con perspectivas de enfoques ampliados en la contratación de enfermeros *gays*, con la intención de formar equipos que puedan desarrollar la autonomía del sujeto y reflejar un acercamiento y un intercambio de ideas más efectivo, son puntos a considerar dentro de las instituciones, así como el estímulo para ofrecer disciplinas específicas en la licenciatura y la maestría en una perspectiva curricular más diversa, fluida y cercana al contexto actual, ya que la formación todavía se centra en la heterosexualidad.

## CONSIDERACIONES FINALES

A pesar de los avances registrados en las políticas afirmativas y en los documentos orientativos existentes en el contexto brasileño, los obstáculos para la eficacia de los cuidados en Enfermería Afirmativa como apoyo a la expresión de la orientación sexual de los hombres mayores *gais* se derivan de la conjunción de negligencias, carencias y ausencias en el campo práctico de los cuidados. La ausencia de enfoques inclusivos en los planes de estudio, el silencio sobre la sexualidad y los tabúes relacionados con el cuerpo de las personas mayores justifican la idea de crear una tendencia asistencial integrada en la política de acción afirmativa como forma de reducir los prejuicios sociales e institucionales y aumentar la adhesión de estos hombres a los servicios de salud.

Es necesario configurar prácticas congruentes con las necesidades de estos hombres para velar por su dignidad en todos los ciclos del desarrollo, preservándolos de cualquier trato inhumano, violento, aterrador, vejatorio o vergonzoso a medida que envejecen.

Se espera que, en el futuro, sea posible una sistematización técnica y científica más estructurada e institucionalizada para que el juicio clínico de los problemas que suscitan respuestas humanas se ejecute mejor y permita transiciones saludables de estos personajes sociales con maestría. Después de todo, cuidar de la salud del hombre gay de edad avanzada es posible basándose en la comprensión de sus demandas y no solo en el cumplimiento de los deberes, sino en la comprensión colectiva de la importancia de ofrecer protección.

Los enfermeros necesitan desarrollar estudios orientados a la comprensión de la salud de los hombres mayores *gays*, ya que este es un hecho que ha limitado la discusión de esta producción de manera más dirigida, ya que la mayoría de los estudios son generalizados e involucran a toda la población de minorías sexuales, de género, de identidad y de expresión de género. Por lo tanto, es necesario delimitar el público que se desea explorar, ya que las particularidades tienden a ser diferentes, así como realizar investigaciones que midan las variables de salud mental y los retos sociales relacionados con la homosexualidad masculina, específicamente, ya que son temas que están asociados.

## DISPONIBILIDAD DE DATOS

Todo el conjunto de datos que respalda los resultados de este estudio se ha publicado en el propio artículo.
